# Comparable human reconstitution following Cesium-137 versus X-ray irradiation preconditioning in immunodeficient NOG mice

**DOI:** 10.1371/journal.pone.0241375

**Published:** 2020-10-29

**Authors:** Anna Halling Folkmar Andersen, Stine Sofie Frank Nielsen, Rikke Olesen, Jakob Le Fèvre Harslund, Ole Schmeltz Søgaard, Lars Østergaard, Paul W. Denton, Martin Tolstrup

**Affiliations:** 1 Department of Infectious Diseases, Aarhus University Hospital, Aarhus, Denmark; 2 Department of Clinical Medicine, Aarhus University, Aarhus, Denmark; 3 Department of Biomedicine, Aarhus University, Aarhus, Denmark; 4 Department of Biology, University of Nebraska at Omaha, Omaha, Nebraska, United States of America; University of Cambridge, UNITED KINGDOM

## Abstract

Humanized mouse models are used extensively in research involving human pathogens and diseases. However, most of these models require preconditioning. Radio-active sources have been used routinely for this purpose but safety issues have motivated researchers to transition to chemical or X-ray based preconditioning. In this study, we directly compare 350 kV X-ray and Cs-137 low-dose precondition of NOG mice before human stem cell transplantation. Based on flow cytometry data, we found that engraftment of human cells into the mouse bone marrow was similar between radiation sources. Likewise, human engraftment in the peripheral blood was comparable between Cs-137 and three different X-ray doses with equal chimerization kinetics. In primary lymphoid organs such as the thymus and lymph nodes, and spleen, liver and lung, human-to-mouse chimerization was also comparable between irradiation sources. Development of different CD4 and CD8 T cells as well as these cells’ maturation stages, i.e. from naïve to effector and memory subsets were generally analogous. Based on our results, we conclude that there are no discernable differences between the two sources in the low-dose spectrum investigated. However, while we encourage the transition to X-ray-based sources, we recommend all research groups to consider technical specifications and dose-finding studies.

## Introduction

Irradiation of immune-deficient mice and subsequent transplant of stem cells is frequently used to develop humanized mice. Historically, cesium (Cs-137) and other radioactive sources have been used for irradiation. However, some researchers have already experienced benefits of switching to an X-ray based irradiation device [1]. These benefits include the fact that X-ray machines can be more affordable and require less facility security compared to Cs-137 sources [2].

Studies have been conducted comparing Cs-137 to X-ray for whole-body myeloablation in non-radiation, immunocompetent mouse strains [2–4] and on the effects of irradiation of stem cells before engraftment [5]. However, there is currently very limited research comparing the effects of using either Cs-137 or X-ray irradiation of immune-deficient mice for the purpose of performing stem cell transplants. Additionally, many studies have been done sequentially and not in parallel or conducted at different research institutions [5–7]. Moreover, there is little information about the lethal effects to the animals and the level of tissue scaring comparing different X-ray voltages in immunodeficient mice, an important detail given the varying radiosensitivity phenotypes inherent to distinct immunodeficient mouse strains [8]. In general, higher energy decreases the attenuation through the target tissue [9]. This means that machines delivering higher peak energy generally produce a more uniform dose with total body irradiation [9]. Since X-ray irradiators generally have lower peak kilovoltage (kVp) than radiation from Cs-137 decay (e.g. 350 kVp compared to Cs-137 662 keV) the output from an X-ray irradiator can be more variable than from a Cs-137 source. Thus, X-ray irradiation outcomes are more dependent on the energy, dose distributions, depth-dose, filtration of beam, etc. of the specific X-ray equipment being utilized. In the host bone marrow niche, the destruction and mobilization of the mixed cell population is critical for successful transplant. Moreover, research using higher doses implies that this niche is comprised of a large number of hypersensitive, modestly radiosensitive and resistant cells [3]. In essence, a relative biological effect (RBE) of around or above 1 is assumed for X-ray vs Cs-137 but differs between organs and in general estimated from studies which cannot be directly translated to ours [3, 4, 10]. Depending upon the experimental question, achieving the same biological effect from X-ray or Cs-137 is not necessarily associated with a one-to-one dose substitution or a simple conversation factor such as the RBE. This can complicate the process of switching from a Cs-137 source to an X-ray device and thus requires in depth optimization to achieve comparable results. Yet, despite the complications created for researchers, many institutions are switching radiation sources to increase safety. These decisions, while laudable and understandable, often put researchers in a position of having to match historical data and protocols to new experimental designs.

We therefore planned a series of experiments to take advantage of a scheduled replacement of our Cs-137 irradiator with a new X-ray-based irradiation system to enable direct comparison of the two irradiation sources. Specifically, we and others at our institution (Wittenborn&Hagert, 2020) performed an extensive head-to-head comparison in the non-lethal, low dose spectrum using stem cell-derived humanized mice models reported herein, as well as the higher radiation doses utilized to precondition non-radiosensitive and immunocompetent strains (Wittenborn&Hagert, 2020). The primary objective of our radiobiology study was to investigate the feasibility of using a newly purchased 350-kV X-ray unit (MultiRad350 X-RAD 350 kV X-Ray Irradiation system (Precision X-Ray Inc, Richland, WA)) compared to the retiring Cs-137 source used previously for the bioengineering of stem cell-transplanted humanized mice. This machine was chosen from available options to replace our Cs-137 source because it provided the ability to integrate and replace X-ray tube-shielding units to achieve the most uniform dosing possible. More specifically, we investigated whether comparable doses of X-ray radiation could efficiently precondition immunodeficient mice and allow for sufficient human donor bone marrow reconstitution in a manner that was analogous to our results with the Cs-137 source. We directly compared human reconstitution outcomes following varying doses of X-ray preconditioning to our established 75 cGy (0.75 Gy) Cs-137 protocol [11, 12].

We tested this X-ray irradiation system using three different doses (0.58 Gy, 0.75 Gy and 0.92 Gy) and compared them to our standard 0.75 Gy Cs-137 dose to aid in choosing the optimal dose for future studies using an X-ray based source. We compared the differences between groups in human chimerization levels (e.g. reconstitution with T and B lymphocytes and myeloid cells) in the peripheral blood and tissues.

## Materials and methods

### Dosimetry

A Gammacelle 2000 RH Cs-137 irradiator (Model AK, Risø, Denmark) has been used in our research department (Aarhus University, Department of Biomedicine).The output from the Cs-137 source was confirmed using a thermoluminescent dosimeter (TLD) phantom that detects radiation placed in the middle (8 cm above the bottom) of the designated radiation container, and approximately in the center of the container diameter (12 mm depth). This is directly in front of the radioactive source and where mice will be placed during irradiation. Based on this calibration, doses were determined with a maximum of 2% variation. Mice received a dose of 0.75 Gy from of the radioactive source during a total of 19.18 secs in the radiation chamber.

The MultiRad350 X-RAD 350 kV X-Ray Irradiation System (Precision Inc) is a self-contained X-ray irradiation system designed for use in biology and medical research. The system utilizes a Thoraeus filter (0.75 mm Tin(Sn), 0.25 mm Copper(Cu) and 1.50 mm Aluminum(Al)) (SnCuAl) to minimize the exposure to unwanted low-energy photons, which can be damaging to animal tissues. Dosimetry and irradiation of animals were performed using settings of 350 kV, 11.4 mA, and targets placed 37 cm from the X-ray tube giving an irradiation field size of 25.6 cm. Dosimetry was performed using Accu-Gold+ sensor (Radcal, Monrovia, CA, USA) and a 10X6-6 chamber (Radcal, Monrovia, CA, USA)

### Ethics statements

Umbilical cord bloods were obtained via anonymous donation under informed written consent from the mother. Animal experiments were conducted under animal license 2017-15-0201-01312 approved by the Danish Animal Experiments Inspectorate. Mice were housed in individually ventilated cages (IVC) in a 12-hrs dark:light cycle with temperature 22°C ±2°C, and free accessibility to acidified water and autoclaved food. Animal technicians performed daily welfare monitoring and weekly body weight measurements.

### Isolation of stem cells

Human stem cells, defined by expression of hCD34, were enriched from umbilical cord blood obtained during caesarean section births of females. Briefly, human stem cells were isolated using EasySep™ Human Cord Blood CD34 Positive Selection Kit II (Stemcell, Vancouver, Canada) column system. The isolated cells were above 85% CD34+ with less than 1% T cell contamination as determined by flow cytometry as previously shown [12] (S2 Fig). Cells were cryopreserved in liquid nitrogen until transplantation.

### Irradiation and stem cell transplantation

**Fig 1:**
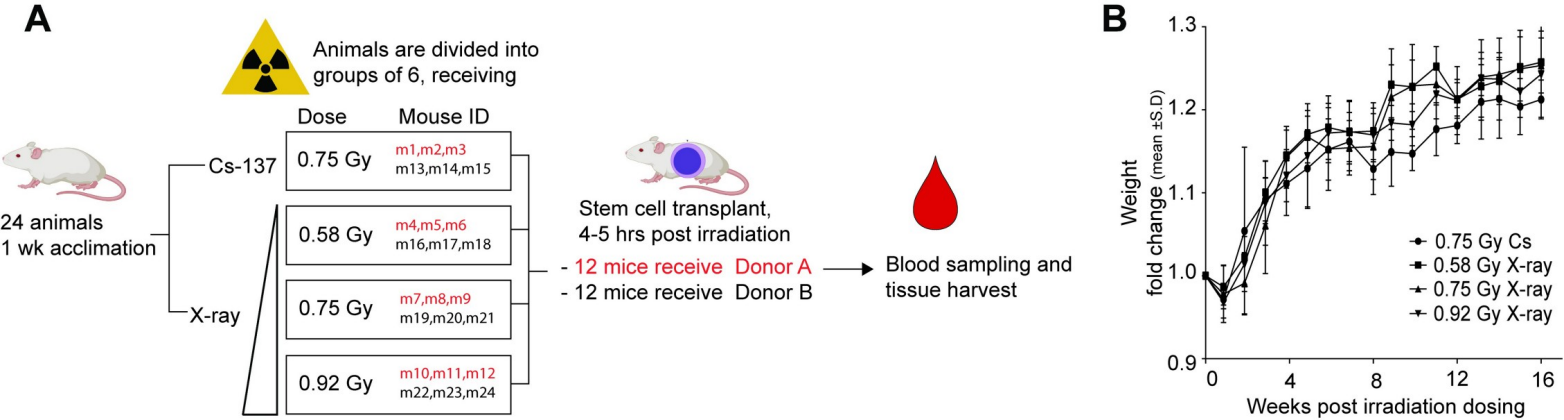
Experimental layout and mouse weight development. (A) Overview of the experimental plan, 24 animals were randomized into 4 irradiation groups. One group received the standard dose in our lab for Cesium-based preconditioning (0.75 Gy of Cs-137 radiation). The remaining mice were divided into three groups that receive either 0.58 Gy, 0.75 Gy or 0.92 Gy of X-ray-derived radiation. The vertical triangle (◿) illustrates increasing doses of X-ray-derived radiation. In each group 3 of the 6 mice received stem cells from donor A, the remaining 3 from donor B. (B) For each group, the starting weight of the individual mice (the same week, pre-engraftment to irradiation (p.e.) were set to the value 1). The recorded weight for the following week is presented as the fold-change from this starting weigh for each specific mouse. Each group is the fold changes for all the mice in each group, presented as mean ± S.D. Weight is an overall estimate of health status of the mice. The mice were exposed 1 or 2 days after p.e. (week 0) weight data were collected.

The purpose of these experiments was to compare the effects of irradiation by a Cs-137 source and an X-ray machine. We used female 5–6 weeks old radiation-sensitive NOD.Cg-Prkdcscid Il2rgtm1Sug/JicTac (Taconic, Silkeborg, Denmark) mice, a strain extremely well suited for bone marrow and stem cell transplants. 12 mice received the cells from one human donor (Donor A) on one day, and another 12 mice received stem cells from a different human donor (Donor B) on the following day. To reduce the potential for distance variability within the Cs-source irradiation chamber, mouse “free moving space” was reduced by padding holding containers with sterile gauze. During the X-ray irradiation, mice were placed in sterile chambers in a standard, sterilized pie cage. Here, mice were able to move towards and away from the center to the periphery of the pie cage. Each mouse received approximately 7.5 x 10^4^ thawed hCD34+ cells suspended in 200 μL RPMI (Biowest, Nuaillé, France) by intravenous injection into the tail vein using a 30G needle 4–5 hrs after irradiation. The experimental design is presented in **Fig 1A**.

### Flow cytometry assessment of immune cell phenotype in peripheral blood

Beginning 12 weeks after transplantation of human stem cells, human/mouse chimerization level in the peripheral blood was assessed at the indicated time points. For each blood sample, approximately 60 μL of blood was collected into 1.5 mL PCR-approved microcentrifuge tubes containing 10 μL of 0.5 M EDTA (Life Technologies, Carlsbad, CA, USA) pH 7.4 to prevent coagulation. Blood was transferred to 5 mL round-bottom tubes and incubated with 5 μL of Human TruStain FcX™ (Biolegend, San Diego, CA, USA) for 10 mins at room temperature. The blood was incubated for 15 minutes at room temperature with the following panel of fluorescently labelled mouse anti-human antibodies obtained from either BioLegend (BL) or BD Biosciences (BD): CD4 (clone SK3) BUV496 (BD564652), CD8 (clone RPA-T8) BV421 (BD562428), CD3 (clone OKT3) FITC (BL217306), CD19 (sj25c1) PE-Cy7 (BD557835) and CD45 (clone 2D1) APC (BL368511). We have validated internally that there is cross-reactivity of the anti-human CD45 antibody towards mouse cells expressing mCD45. Red blood cells were subsequently lysed using FACS Lysing solution (BD Bioscience, San Jose, CA, USA) according to manufacturer’s recommendations. The resultant white blood cells (WBCs) which were analyzed using a BD LSRFortessa™ flow cytometer (BD Biosciences, San Jose, CA, USA). After FSC vs SSC and singlet discrimination, the peripheral engraftment percentage was calculated for overall human leucocytes (based on expression of hCD45) out of total detected WBCs. The overall human leucocyte percentage was further analyzed to determine the percentage of lymphocytes (T and B cells) based on expression of hCD3 and hCD19.

### Harvest of mice for assessment of human immune reconstitution in tissues

Mice were sacrificed in the time window 23 to 29 weeks post-transplant, with mice from the same irradiation group killed both in the beginning and in the end of the time interval. This ensured that any changes observed between the groups were not due to progression of the graft development. Importantly, at this stage (over 23 weeks) the graft is considered relatively “stable”, based on our own lab empirical data and published data [13, 14].

Lymph nodes (mesenteric, superficial inguinal, brachial, axial and cervical), spleen, thymus, bone marrow, liver and lung were resected. In brief, single cell suspensions were isolated from tissues by the following procedures: Tissue was harvested into cold suspension media ([PBS supplemented with 5 g/L bovine serum albumin (Sigma-Aldrich, St. Louis, MO, USA); penicillin/streptomycin (Biowest, Nuaillé, France); and DNase (Sigma-Aldrich, St. Louis, MO, USA)) Tissues were subsequently processed essentially as previously described [15, 16]. In brief, lymph nodes, spleen, thymus were gently disrupted using a plunger from the 5 mL syringe onto a 70 μm cell strainer (Corning Life Sciences, NY, USA), passing individual cells through the strainer. Tissue remnants and the strainer were washed with suspension media and the flow-through was centrifuged (5 mins; 300xg; 4°C), supernatant was removed and the cell pellet resuspended in 10 mL of 1x RBC lysis buffer (BioLegend, San Diego, CA, USA). The cell suspension was incubated for 10 mins at 4°C. After red blood cell lysis, cells were pelleted, washed in suspension media and resuspended in 1 mL suspension media for viable cell counting using trypan blue exclusion. Cells were afterwards processed for flow cytometry as described above.

Liver and lung tissues were minced and digested in an enzyme cocktail containing DNAse I (Sigma-Aldrich, St. Louis, MO, USA), elastase (Sigma-Aldrich, St. Louis, MO, USA), hyaluronidase (Sigma-Aldrich, St. Louis, MO, USA), collagenase (Sigma-Aldrich, St. Louis, MO, USA) and RPMI (Biowest, Nuaillé, France) with constant agitation at 37°C for 60 mins. After digestion, and single cell preparation as described above, liver and lung cells were collected at the interface of a 40% to 70% Percoll (Sigma-Aldrich, St. Louis, MO, USA) density separation centrifugation. Cells were washed once in suspension media and resuspended in 1 mL suspension media for viable cell counting using trypan blue exclusion and processed for flow cytometry.

### Flow cytometry assessment of immune cell phenotypes in tissues at time of harvest

The following flow cytometry antibodies obtained from either BioLegend (BL) or BD Biosciences (BD) was used in one panel for isolated tissue WBCs: CD3 (clone SK7) BUV395 (BD564001), CD4 (clone SK3) BUV496, (BD564652), CD14 (clone M5E2) BUV737 (BD612763), CD38 (clone HIT2) BV421 (BD562444), CD33 (clone P67.6) BV605 (BL366611), CD197(CCR7) (clone G043H7) BV785 (BL353230), CD45RA (clone HI100) FITC (BD555488), CD16 (clone 3G8) PerCP-Cy5.5 (BL302028), HLA-DR (clone G46-6) PE (BD555812), CD19 (clone sj25c1) PE-Cy7 (BD557835), CD69 (clone FN50) APC (BL310910), CD8 (clone RPA-T8) APC-R700 (BD565165), and CD45 (clone 2D1) APC-H7 (BD560178). In tissues, cells are gated into subsets based on the flow cytometry gating strategy presented in S1 Fig.

### Statistical analysis

Flow cytometry data was analyzed in FlowJo v10.6.2 (Treestar, Ashland, OR, USA) and statistical analysis was performed in Graphpad Prism (Graphpad Software, San Diego, CA, USA). Suitable for small datasets without assuming normal distribution, Mann-Whitney U tests with Dunn’s multiple comparisons was used to compared cross-sectional results from each experimental irradiation group, or multiple t-tests using Holm-Sidak correction for multiple comparisons. Data was deemed statistical significant when P < 0.05 (*) and P < 0.01 (**)

## Results

### Animal health

Mice weights were recorded weekly, starting from the week they were received into the facility. As presented in **Fig 1B**, all groups of mice experienced a small weight loss following the irradiation procedure. The subtle initial weight loss was followed by steady weight gains indicating no sustained adverse effects of the radiation regardless of the source or exposure level [17, 18]. All groups showed similar weight development in weight over time, indicating similar health status of the mice after the different irradiation procedures and doses.

Upon necropsy, graft-vs-host-disease (GVHD) symptoms were found in three mice. The macroscopic symptoms included enlarged spleen and lymph nodes. Other typical symptoms of GVHD are inflamed skin, hair loss, weight loss, stereotypical behavior or reduced activity levels [19, 20]. We did not observe any of these other typical symptoms in any of the mice with enlarged lymphoid organs, which led us to interpret that the three mice were early in the GVHD progression. The three mice, m1, m2 and m3 were all in the same experimental group (irradiated with 0.75 Gy Cs-137 and receiving stem cells from Donor A). We attribute the onset of GVHD to a combination of donor and source of irradiation given that none of the mice (m4-m12) receiving the same number of human stem cells from the same human donor (but after X-ray preconditioning) developed any detectable GVHD symptoms.

### Donor-dependent kinetics of human-mouse chimerization in the peripheral blood

To maximize the longitudinal information that could be obtained from our cohorts, no mice were sacrificed for tissue analyses prior to 23 weeks post-transplant. Cross-sectional analyses of the grafts in various tissues occurred between weeks 23 and 29 post-transplant. However, the cross-sectional analyses provide powerful data that facilitate nuanced comparisons of the human engraftment of both inductive and effector immune sites throughout the body following radiation and human stem cell transplantation.

Flow cytometry analysis revealed early repopulation of cells from the B cell lineage (CD19) in the peripheral blood (PB) of all mice, and delayed T cell lineage reconstitution (CD3), as anticipated based upon lab empirical, as well as, published data [13, 14]. In general, B cells repopulate the peripheral circulation earlier following transplantation, because these cells derive and mature in the bone marrow [21], whereas T cells levels rise slower because immature thymocytes first need to leave the bone marrow and migrate to the thymus. In the thymus, T cells mature and undergo positive and negative selection before emigrating and entering peripheral circulation.

**Fig 2:**
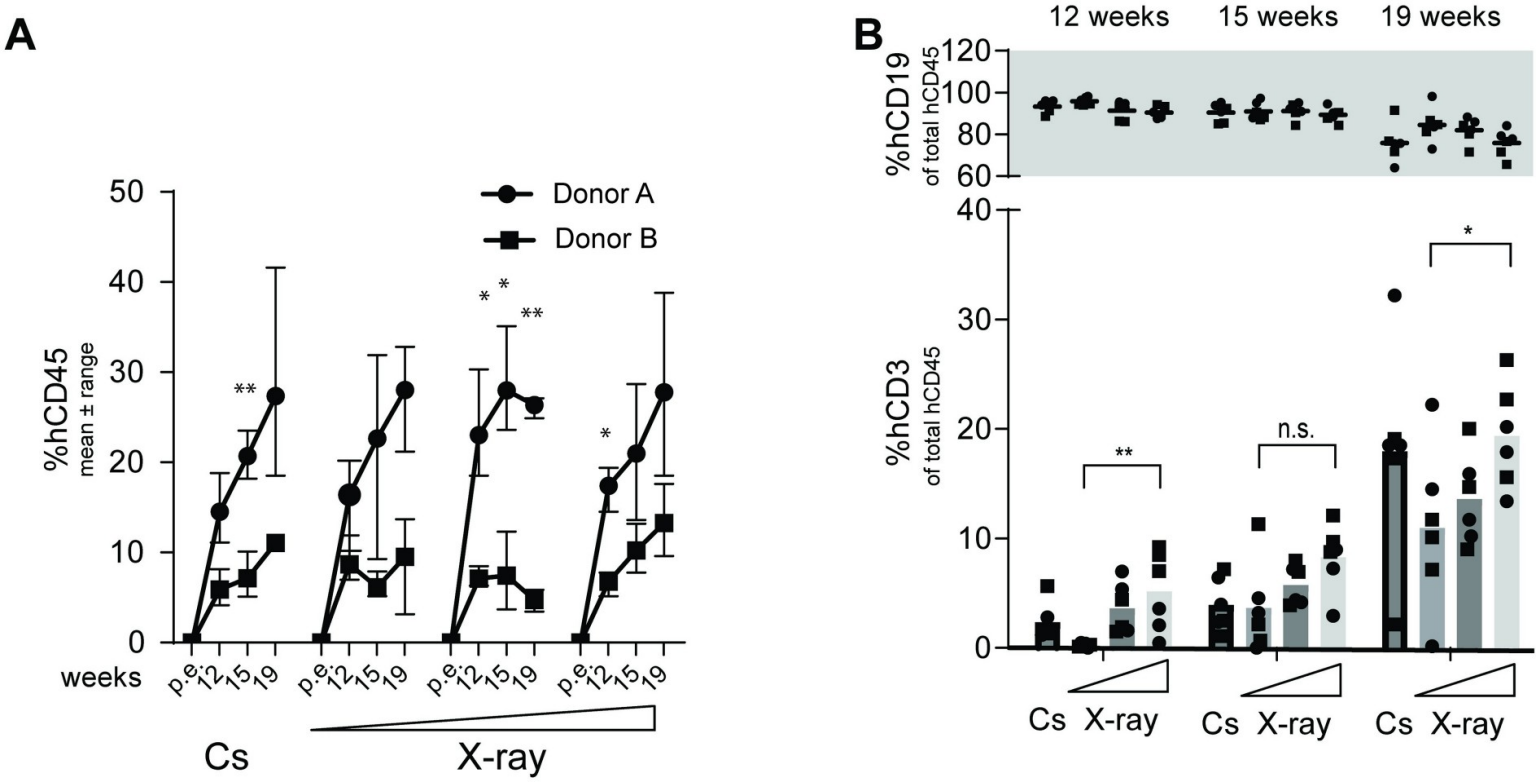
Human chimerization in peripheral blood depicted for each donor as well as for each source of irradiation and dose. (A) Percentage (mean ± range) of peripheral cell chimerization (based on fraction of all WBCs detected) (⚫Donor A, ⬛Donor B). Statistical significant differences between donors for each time points were calculated using multiple t-tests with Holm-Sidaks correction for multiple comparisons. Asterisks are depicted above significant differences. (B) Percentage (mean ± range) of either peripheral B cells (top panel) or T cells (bottom panel) (based on fraction of WBCs that express human CD45) presented as a mean of all 6 mice in each radiation group, the two donors combined (⚫Donor A, ⬛Donor B). Relevant statistical comparisons between irradiation groups using Mann-Whitney U tests are depicted above bars.

Variation in peripheral blood human cell reconstitution was observed between mice from distinct human donors (**Fig 2A and 2B**). Significant differences were found between the total group of 12 mice derived from Donor A versus Donor B at week 19 regarding percent human CD45 (Mann-Whitney, P<0.0001). The level of T cells was not significantly different between the human donors, indicating a comparable biological progression of cell differentiation (Mann-Whitney, P = 0.5996). The 6 mice receiving 0.58 Gy X-ray (independent of donor) had significantly lower proportion of T cells at the 12- and 19-week time point compared to the 0.92 Gy X-ray group (Mann-Whitney, P = 0.0022 and P = 0.0411, respectively) (**Fig 2B**).

### Comparable bone marrow engraftment between Cs-137 and X-ray-irradiated mice

**Fig 3:**
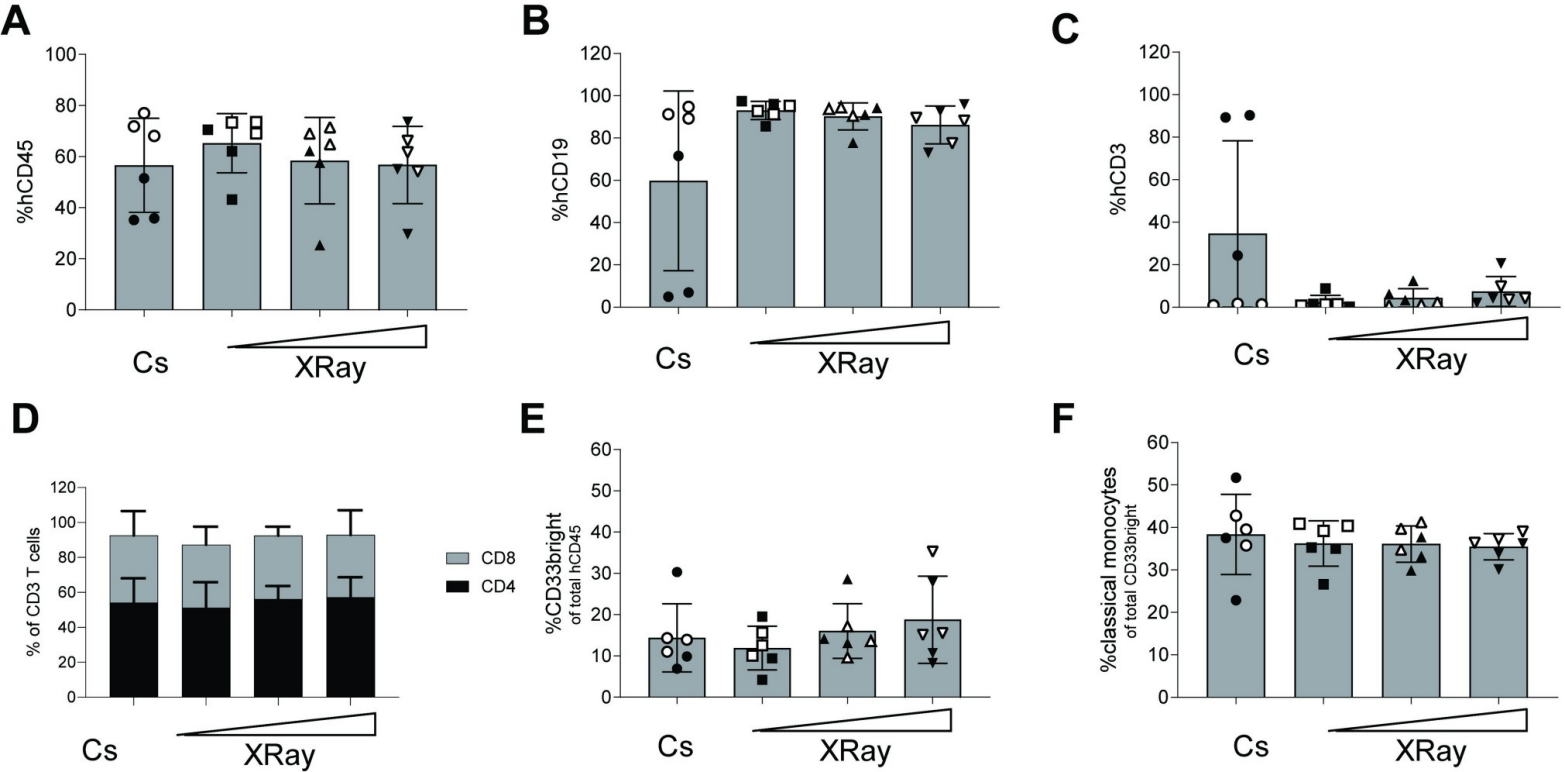
Bone marrow in NOG mice humanized following either irradiation from a Cs source or an X-ray device. (A) Bar graph for each irradiation group showing the percentage of human cells, based on expression of hCD45 out of total WBCs identified in a forward/side-scatter flow cytometry gating plot. (B) Bar graph of the frequencies of human B cells based on expression of surface hCD19. (C) Bar graph of the frequencies of human T cells based on expression of surface hCD3. (D) Bar graphs of frequencies of human T cell subsets based upon expression of surface CD4 or CD8 presented in (C). (E) Bar graphs presenting myeloid cells based on expression of CD33 out of total hCD45. (F) Classical monocytes based on expression of CD14 and lack of CD16-expression (CD14+CD16neg) within the myeloid cells population in (E). Each dot represents one mouse (6 mice per group) presented with mean ± S.D.The horizontal triangle (◿) illustrates increasing doses of X-ray-derived radiation. Stem cell Donor A-derived mice are full circles (●) and Donor B-mice empty circles (○).

At harvest, we assessed the bone marrow reconstitution via flow cytometry. We found that mean percentages of humanization were around 60% based on hCD45 expression out of total WBCs for all four groups. There were no significant differences (P > 0.05, Mann-Whitney test, with Dunn’s multiple comparisons) between X-ray and Cs-137 sources in terms of human to mouse percent chimerization (**Fig 3A**). Furthermore, we did not detect an impact of the different X-ray doses. The major compartment of the human cells are CD33neg, non-myeloid cells. These were almost exclusively of human B lineage, based on expression of CD19 (**Fig 3B**), except for m1, m2, m3 (which showed signs of GVHD). All other mice, regardless of preconditioning regimen or human donor, had none-to-10% T cells in their bone marrow (**Fig 3C**). When observed, T cells levels were split into approximately equal parts CD4+ T cells and CD8+ T cells (**Fig 3D**), with no significant differences between any of the irradiation groups. Of the hCD45+ cells in the bone marrow, approximately 15% of these cells were CD33bright myeloid cells (**Fig 3E**). For all four groups, approximately 38% of these myeloid cells were classical monocytes, based on expression of CD14 without concomitant expression of CD16 (**Fig 3F**). Therefore, we conclude that bone marrow engraftment was equal irrespective of low-level irradiation source.

### Mouse thymi and lymph node tissues are almost exclusively comprised of human white blood cells

**Fig 4:**
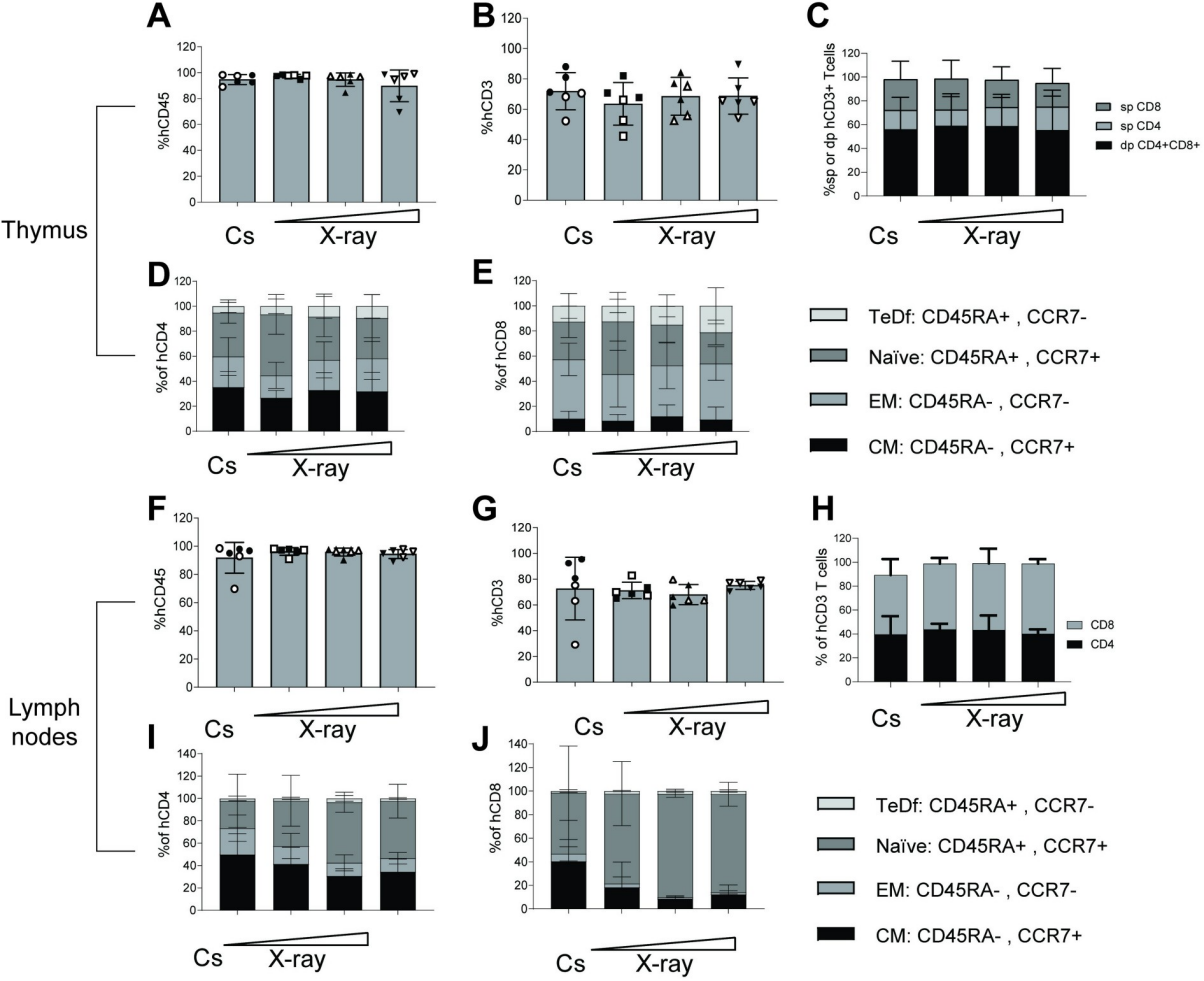
Thymopoiesis and lymph node tissues in NOG mice humanized following either irradiation from a Cs source an X-ray device. (A) Bar graph for each irradiation group showing the percentage of human cells based on expression of hCD45 out of total WBCs identified in a forward/side-scatter flow cytometry gating plot in thymi. (B) Bar graph of the frequencies of human T cells based on expression of surface hCD3 in thymi. (C) Bar graphs of the human CD3 T cells identified into either CD4 single positive (sp), CD8 sp or CD4CD8 double positive (dp) in thymi. (D)+(E) Distribution of either CD4 sp or CD8 sp T cells into four maturation stages, i.e. naïve, central memory (CM), effector memory (EM) or terminally differentiated (TeDf) T cells based on surface expression of human CCR7 and CD45RA. (F) Bar graph for each irradiation group showing the percentage of human cells based on expression of hCD45 out of total WBCs identified in a forward/side-scatter flow cytometry gating plot in lymph nodes (mesenteric, superficial inguinal, brachial, axial and cervical). (G) Bar graph of the frequencies of human T cells based on expression of surface hCD3 in lymph nodes. (H) Compartmentalization of the human CD3 T cells identified into either CD4 sp or CD8 sp in lymph nodes (I+J) Data bars of the frequencies of CD4 and CD8 T cell subsets, respectively based on expression of CD45+ and CCR7-. Each dot represents one mouse (6 mice per group). Data is presented with mean ± S.D. The horizontal triangle (◿) illustrates increasing doses of X-ray-derived radiation. Stem cell Donor A-derived mice are full circles (●) and Donor B-mice empty circles (○).

Human intra-thymic T-cell development in the irradiated, transplanted mice was also assessed at necropsy. Thymus tissues in irradiated NOG mice are under-developed until after human stem cell engraftment which generates T cell precursors that migrate from the bone marrow to populate the thymus and undergo thymopoiesis. Thymocytes were immunophenotyped using flow cytometry analysis for their major developmental subsets, based on expression of CD4+ and CD8+ (i.e.“double positive, dp” or, “single positive”, CD4sp, CD8sp). Human chimerization in all thymus tissues examined was determined based on expression of hCD45 and was found to be around or above 90% of total WBCs (**Fig 4A**). As expected, these human thymocytes were primarily CD3+ T cells (**Fig 4B**). When comparing proportions of CD4sp vs CD8sp cells, we observed no difference in frequencies between any of the irradiation groups (**Fig 4C**). In addition, no statistical significances were found when comparing frequency of CD4+CD8+ dp cells. This clearly indicates consistent thymopoiesis occurring in all mice regardless of irradiation preconditioning regimen or human donor.

Differences were found between groups when comparing the mature helper and cytotoxic T cell subsets based on expression of CCR7 and CD45RA. These markers are used to distinguish naïve, effector memory (EM), central memory (CM), and terminally differentiated (TeDf) subsets. We observed a tendency towards higher frequencies of naïve cells and fewer CM cells in the 0.58 X-ray group compared to all other three groups, particularly for the CD4sp and to some extend CD8sp thymocytes (**Fig 4D and 4E**).

The lymph nodes are secondary lymphoid organs and are important for developing adaptive immune responses. At necropsy, we resected lymph nodes and processed the tissues together into a single suspension of combined lymph node cells for each mouse. Of these lymph node cells close to 100% were of human origin (**Fig 4F**). This was independent of radiation and human stem cell donor. Lymph node cells were primarily of the T-cell lineage (**Fig 4G**) as evidenced by their expression of CD3. These T cell populations were comprised of approximately 40% CD4+ and 40–50% CD8+ T cells (**Fig 4H**). Within the CD4+ T cell compartment, approximately 40% of cells were CM, 40% naïve and the rest primarily EM, and there was a very low frequency of TeDf (**Fig 4I**). The CD8+ T cell compartment in the lymph nodes was predominantly comprised of naïve cells (around 80%) followed by 10% CM (**Fig 4J**) in all mice. The tendency towards more CM in the Cs-137 group in both CD4 and CD8 population was driven by the three mice developing GVHD.

### Comparable immunological phenotypes of human cells between irradiation groups found in spleen, liver and lungs

**Fig 5:**
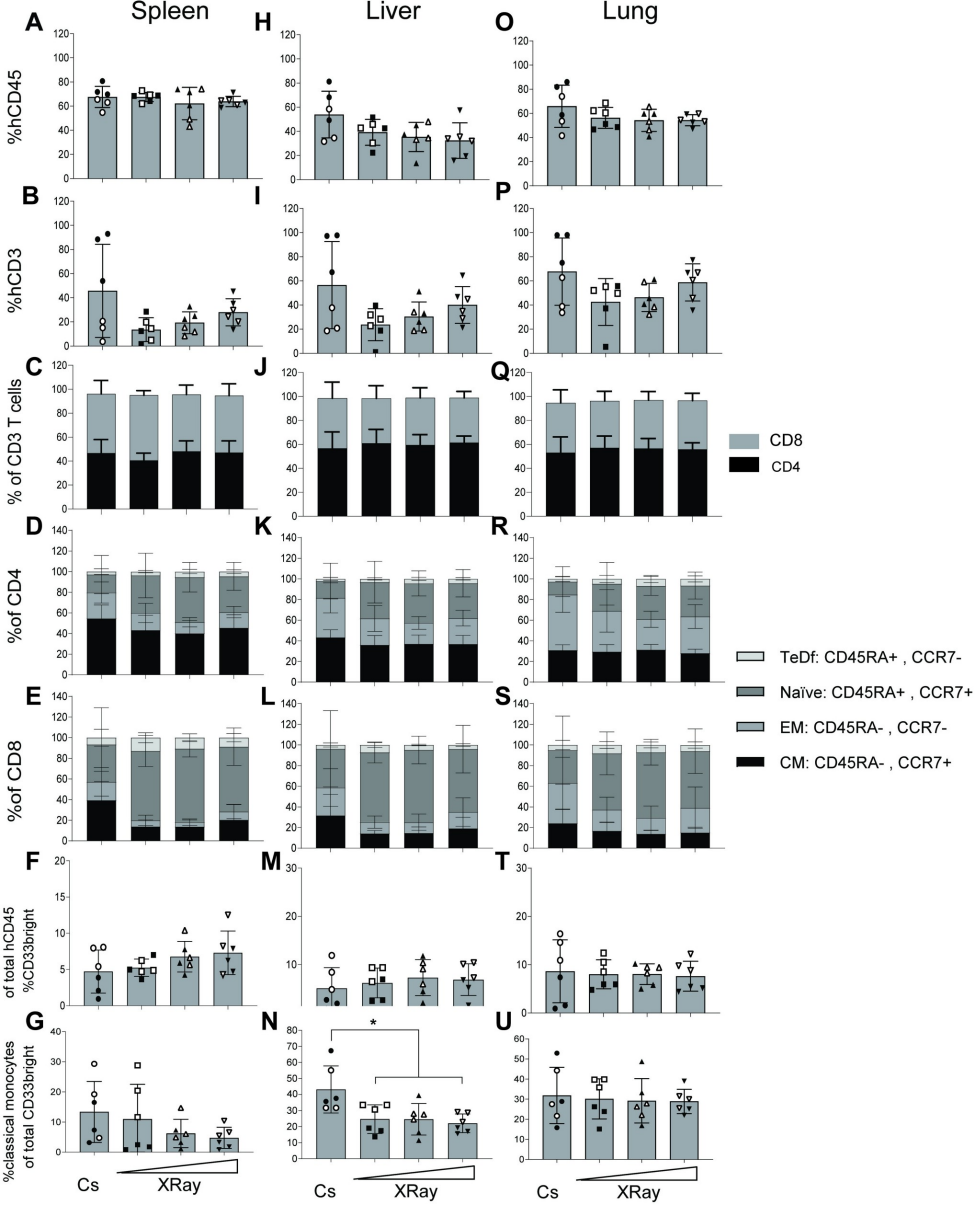
Human cell engraftment in inducer and effector tissues, spleen, liver, lung. (A+H+O) Bar graph for each irradiation group showing the percentage of human cells based on expression of hCD45 out of total WBCs identified in a forward/side-scatter flow cytometry gating plot in spleen, liver and lung, respectively. (B+I+P) Bar graph of the frequencies of human T cells based on expression of surface hCD3 in spleen, liver and lungs, respectively. (C+J+Q) Compartmentalization of the human CD3 T cells identified into either CD4 sp, CD8 sp or CD4CD8 dp in spleen, liver and lungs, respectively. (D+K+R) Distribution of either CD4 sp T cells into four maturation stages, i.e. naïve, CM, EM or TeDf based on surface expression of human CCR7 and CD45RA in spleen, liver and lungs, respectively. (E+L+S) Same as for D+K+R but for CD8 sp T cells. (F+M+T) Myeloid cells based on expression of CD33 out of total hCD45 in spleen, liver and lungs, respectively. (G+N+U) Classical monocytes based on expression of CD14 and lack of CD16-expression based as a fraction of the myeloid cells (F+M+T) in spleen, liver and lungs, respectively. Each data point represents one mouse (6 mice per group). Data is presented with mean ± S.D. Stem cell Donor A-derived mice are full circles (●) and Donor B-mice empty circles (○).

To further assess the engraftment of human cells into immune inductive as well as effector organs, we also analyzed human engraftment levels in the spleen, liver and lungs of mice at necropsy. In the spleen, the percent hCD45 in all four irradiation groups ranged from 40 to 80%. The mean across all groups were 60–70% human, regardless of human stem cell donor or irradiation source (**Fig 5A**). The human cells were primarily of the B-cell lineage based on expression of CD19, and T cells around 20–40% for most mice (**Fig 5B**) except m1, m2, m3 (S1 Table). These mice had infiltration of T lymphocytes into the tissues, another indicative of GVHD [22]. Of CD3+ splenocytes, the distribution between CD4+ T helper cells and CD8+ T cytotoxic cells was approximately 1-to-1 (**Fig 5C**) for all mice. No differences were found between groups when comparing the distribution of CD4-expressing helper T cells into maturation levels. These cells were primarily central memory or naïve CD4+ splenocytes (**Fig 5D**), regardless of human donor and preconditioning regimen. These two cell subsets express CCR7, which along with LFA-1 and L-selectin mediates homing of T cells to secondary lymphoid organs, such as the spleen, via high endothelial venules (HEVs) [23]. Similar outcomes were observed for the CD8+ splenocytes except that m1, m2 and m3 exhibited elevated EM and CM levels relative to all other mice (**Fig 5E**). In the spleen, a small fraction (5–10%) of human CD45-expressing cells were of the myeloid lineage (based on expression of CD33+, a myeloid marker) (**Fig 5F**) fraction was consistent between all four radiation groups. Similar to our findings in the spleen where no notable differences between experimental groups were observed, the liver and lung tissues examined also showed overall consistency in human reconstitution regardless of the preconditioning regimen or human donor used (**Fig 5H–5U**). Thus, we conclude that in inductive and effector sites we found that the X-ray irradiator and Cs-137 irradiator performed similarly for preconditioning the bone marrow of NOG mice for human hematopoietic stem cell transplantation.

## Discussion

This study sought to directly compare the abilities of an X-ray and Cs-137 irradiator to perform bone marrow preconditioning in NOG mice. We compared a sub-lethal dose of X-ray irradiation directly to an established Cs-137 protocol by monitoring peripheral blood chimerization longitudinally as well as long-term tissue engraftment cross-sectionally. Our specific focus was development of human immune cells, particularly T cells. Switching the irradiation source (Cs-137 to X-ray) did not impact the magnitude or kinetics of peripheral human leucocyte reconstitution. In addition, there were no discernible effects of choice of radiation on subsequent differentiation and maturation into human immune cells. Specifically, the doses used in this study did not reveal any major differences between equal doses of radiation from our X-ray system and Cs-137 sources in terms of overall animal health, analyzed by mouse weight progression and organ chimerization.

Irradiation of immunodeficient mice before transplantation enhances hematopoiesis and survival of donor cells and could be influenced by the inherent differenced in physical properties between Cs-137 and X-ray radiation. Besides inducing increased expression of stem cell factor (SCF), which enhances hematopoiesis and survival, the bone marrow ablation acts to destroy actively dividing cells, and can thus affect the survival of essential host cells [24] as well as the quality of the human engraftment. Our experimental design included the use of two distinct human donors to ensure that our conclusions are representative of more than a single human donor. Differences in human reconstitution between donors in these types of studies can be partly attributed to the fact that donor stem cell batches are highly diverse and can be difficult to define based on surface markers [25, 26]. Moreover, three mice (m1-m3) arising from one donor, irradiated with Cs-137 presented with a different human cell constitution. We believe that the presence of a less naïve phenotype in these mice along with the dominance of CD3 T cells in the periphery and organs together reflect a GVHD development its early phases. A subset of committed stem cells found in Donor A could have had beneficial engraftment into the mouse tissue environment, which favored development of host-reactive T cells. Our anecdotal evidence using this donor (a study starting before but concluding concurrently with this study) supports this notion, since 2 out of 6 mice in this other study developed more severe signs of GVHD. These data speak towards implementing routine HLA-typing of the human stem cell donors, since the HLA-type could play a role in the disposition towards GVHD development [20, 27]. However, more extensive studies are needed to truly map the influence of radiation source in combination with donor HLA-types in these mice for humanization as we cannot rule out the possibility that Cs-137 irradiation results in a different human reconstitution quality.

To our knowledge, no similar radiation comparison studies are published using a humanized mouse model, and since different mouse strains respond differently to radiation based on immune competency [28], extrapolating knowledge from previous studies to humanized mouse application can be challenging. Indeed, when comparing our results to studies with higher doses for bone marrow transplantation in immune competent mice, we find that our data do not immediately reflect these, as conflicting data exists to whether, X-ray appears more lethal and less effective compared to Cs-137 in allowing donor bone marrow engraftment [2, 10]. To this end, a parallel study was conducted at our facility, analyzing bone marrow transplantations in non-radiosensitive and immunocompetent strains (Wittenborn&Hagert, 2020). In this study, relative biological effectiveness of X-ray to Cs-137 was also comparable but also revealed dose-dependent toxicities and other biological differences. These nuanced data indicate how outcomes are highly dependent on the choice of X-ray source and accessory equipment.

To ensure continuity between past experiments and future experiments, we therefore sought an X-ray machine that would mimic Cs-137 as much as physics would allow. Newer technologies and shielding strategies have made this goal more attainable. Avoiding lower-energy radiation that do not have the ability to penetrate deep into soft tissue was crucial in our considerations during this transition. Our opinion is that researchers should aim to obtain X-ray equipment that allows for a high peak photon energy. The specific instrument used in this study (MultiRad350 X-RAD 350 kV X-Ray Irradiation system) has a 350 kV-peak energy and with the applied filtering proved to have sufficient radiation power with minimal-to-no significant radiation-induced pathology.

A major strength in this study (and Wittenborn&Hagert, 2020) conducted at our facility was the ability to perform precondition with both radiation sources simultaneously during the brief transition period between irradiators. Access to both machines allowed us to perform head-to-head comparisons using animals that were housed in the same vivarium together for the individual studies. Furthermore, for this study, the setup allowed us to transplant all mice, receiving cells from a single human donor on the same day without having to thaw and prepare cells multiple times. These features reduce confounders and limit introduced experimental variables to the irradiation devices themselves.

We conclude that both X-Ray and Cs-137 sources were able to sufficiently ablate the mouse bone marrow to allow human stem cell engraftment. However, broader understanding of the exact mechanisms in play warrants more in-depth analyses to understand undiscovered physiological responses associated with radiation for development of chimeric models. In summary, we have shown replacing our Cs-137 irradiation with an X-ray-based system allows us to perform experiments using equivalent radiation doses while maintaining the ability to directly compare previous data with future data.

## Supporting information

S1 Fig Overall organ gating strategy based on representative lung tissue. Forward/side scatter was used to gate lymphocytes and myeloid cells. Subsequently, doublets were excluded based on FSC-A/FSC-H. Then, overall human leukocytes were distinguished from mouse cells by hCD45/SSC-A gating. Cells were then separated into CD33neg, CD33+ cells. Of the CD33+ cells, classical monocytes were defined based on CD14/CD16 expression. Of the CD33neg cells, B and T cells were separated based on expression of CD19 and CD3. The CD3+ cells were subsequently separated into CD4+ and CD8+ T cells. These T cells can be separated into naïve, effector memory (EM), central memory (CM) and terminally differentiated (TeDf) based on expression of CCR7 and CD45RA.
(TIF)

S2 Fig CD34 cord blood purity. Stem cell purity assessed by flow cytometry analysis. Acquired events were gated based on total cells (FSC-A/SSC-A), then doublets were discriminated using a FSC-H/FSC-A gate, and depris was excluded based on FSC-A/SSC-A). Next, hCD45+ were gated and presented on (A) and (B) for cells originating from Donor A and B, respectively. Percent CD34+CD3neg and CD3+CD34neg for each donor is presented.
(TIF)

S1 Table Cell population frequencies based on flow cytometry. Data used to produce Figs 3–5 is presented for each mouse. Each mouse in each group is presented horizontally, and groups differ vertically. One can assume that values are matched, such that e.g. the first value in each group derives from the same mouse, and so forth.
(XLSX)
