# Supplementary figures and images for: Comparable human reconstitution following Cesium-137 versus X-ray irradiation preconditioning in immunodeficient NOG mice
Source: PLoS One. 2020 Oct 29;15(10):e0241375. doi: 10.1371/journal.pone.0241375 (PMC7595384; doi:10.1371/journal.pone.0241375)

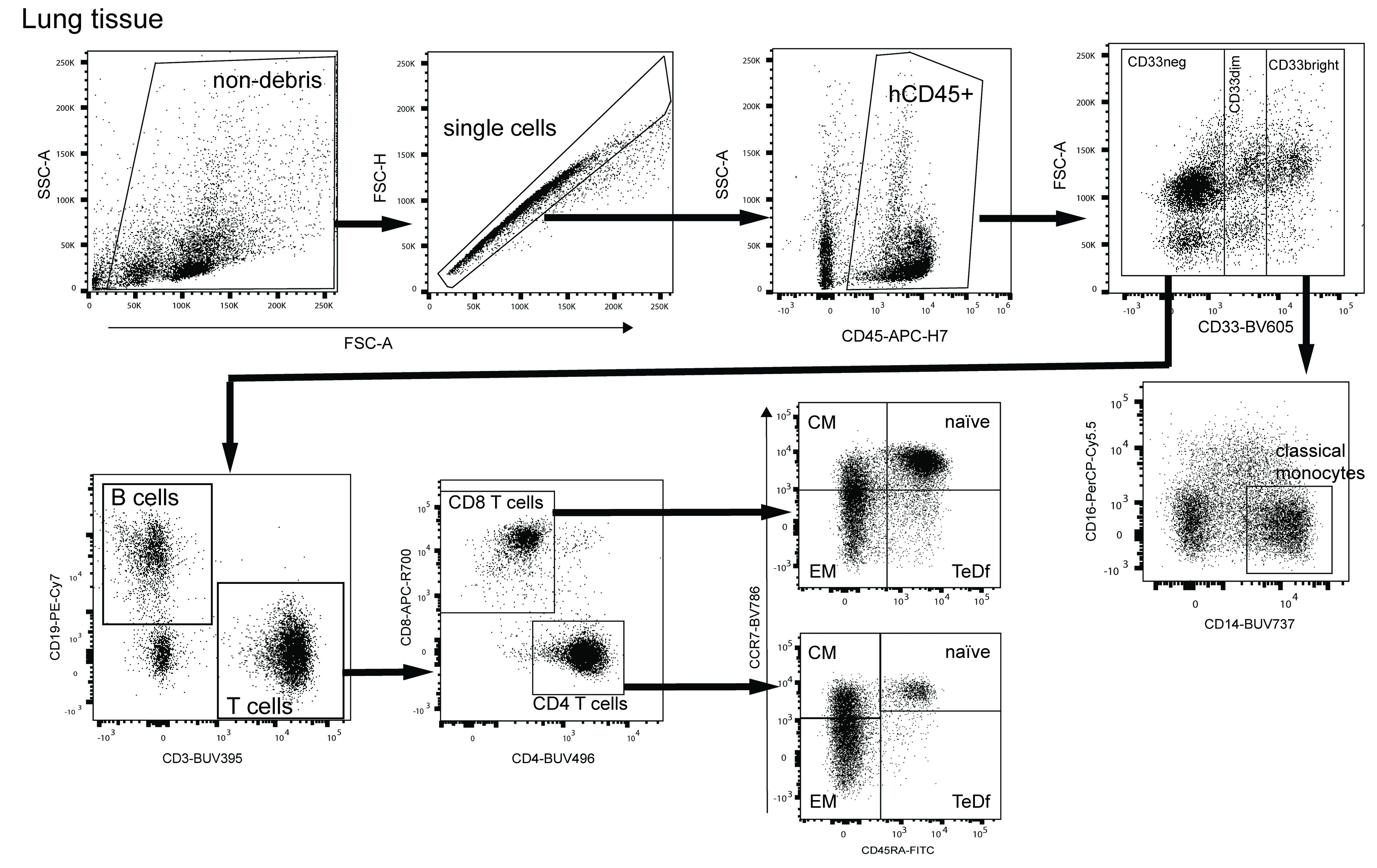

Supplement: S1 Fig — Forward/side scatter was used to gate lymphocytes and myeloid cells. Subsequently, doublets were excluded based on FSC-A/FSC-H. Then, overall human leukocytes were distinguished from mouse cells by hCD45/SSC-A gating. Cells were then separated into CD33neg, CD33+ cells. Of the CD33+ cells, classical monocytes were defined based on CD14/CD16 expression. Of the CD33neg cells, B and T cells were separated based on expression of CD19 and CD3. The CD3+ cells were subsequently separated into CD4+ and CD8+ T cells. These T cells can be separated into naïve, effector memory (EM), central memory (CM) and terminally differentiated (TeDf) based on expression of CCR7 and CD45RA. (TIF) [file pone.0241375.s001.tif]

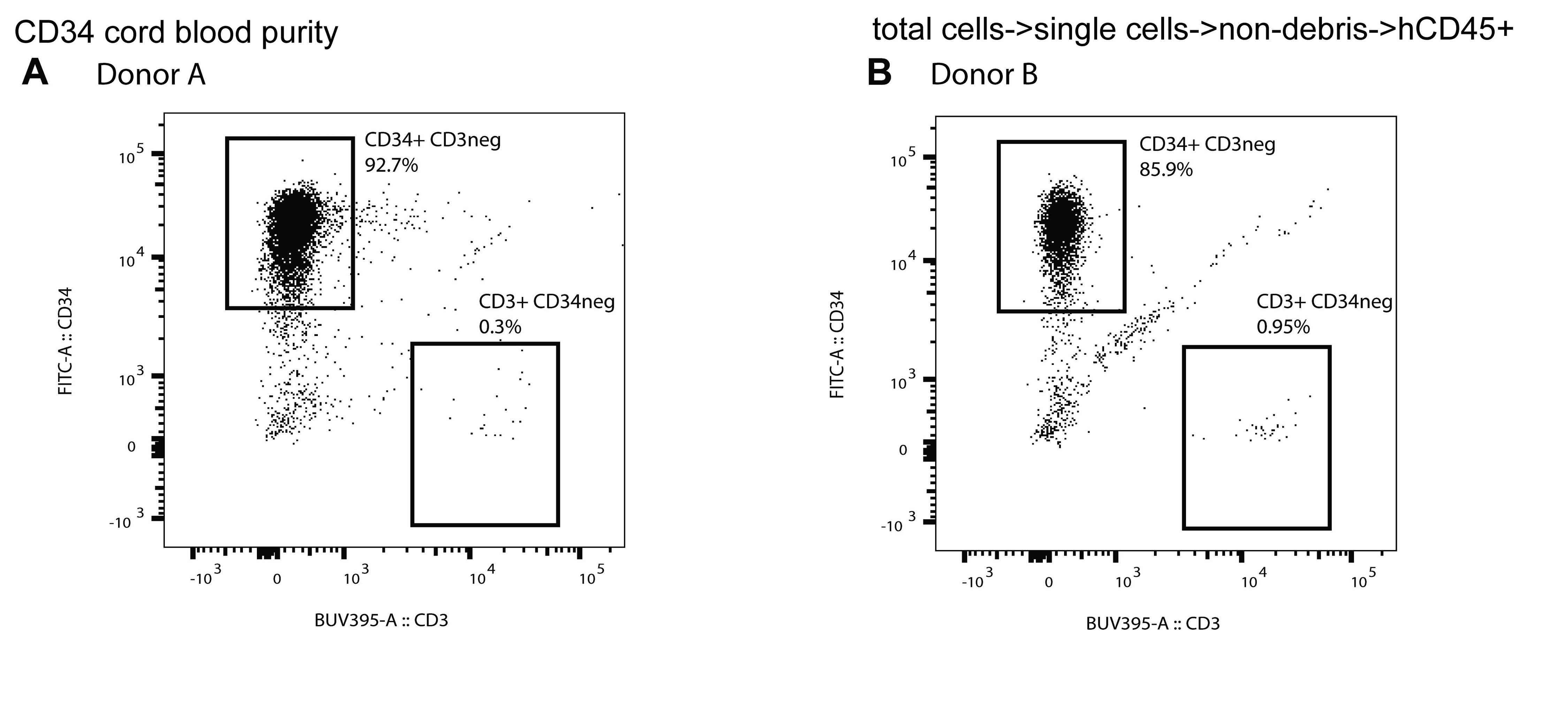

Supplement: S2 Fig — Stem cell purity assessed by flow cytometry analysis. Acquired events were gated based on total cells (FSC-A/SSC-A), then doublets were discriminated using a FSC-H/FSC-A gate, and depris was excluded based on FSC-A/SSC-A). Next, hCD45+ were gated and presented on (A) and (B) for cells originating from Donor A and B, respectively. Percent CD34+CD3neg and CD3+CD34neg for each donor is presented. (TIF) [file pone.0241375.s002.tif]
